# Unravelling the Complexity of Inherited Retinal Dystrophies Molecular Testing: Added Value of Targeted Next-Generation Sequencing

**DOI:** 10.1155/2016/6341870

**Published:** 2016-12-29

**Authors:** Isabella Bernardis, Laura Chiesi, Elena Tenedini, Lucia Artuso, Antonio Percesepe, Valentina Artusi, Maria Luisa Simone, Rossella Manfredini, Monica Camparini, Chiara Rinaldi, Antonio Ciardella, Claudio Graziano, Nicole Balducci, Antonia Tranchina, Gian Maria Cavallini, Antonello Pietrangelo, Valeria Marigo, Enrico Tagliafico

**Affiliations:** ^1^Center for Genome Research, University of Modena and Reggio Emilia, Modena, Italy; ^2^Department of Medical and Surgical Sciences, University of Modena and Reggio Emilia, Modena, Italy; ^3^Institute of Ophthalmology, University of Modena and Reggio Emilia, Modena, Italy; ^4^Medical Genetics Unit, Azienda Ospedaliero-Universitaria di Parma, Parma, Italy; ^5^Department of Life Sciences, University of Modena and Reggio Emilia, Modena, Italy; ^6^Centre for Regenerative Medicine, University of Modena and Reggio Emilia, Modena, Italy; ^7^Ophthalmology, S.Bi.Bi.T. Department, University of Parma, Parma, Italy; ^8^Ophthalmology Unit, Policlinico S. Orsola-Malpighi, Bologna, Italy; ^9^Medical Genetics Unit, Policlinico S. Orsola-Malpighi, Bologna, Italy

## Abstract

To assess the clinical utility of targeted Next-Generation Sequencing (NGS) for the diagnosis of Inherited Retinal Dystrophies (IRDs), a total of 109 subjects were enrolled in the study, including 88 IRD affected probands and 21 healthy relatives. Clinical diagnoses included Retinitis Pigmentosa (RP), Leber Congenital Amaurosis (LCA), Stargardt Disease (STGD), Best Macular Dystrophy (BMD), Usher Syndrome (USH), and other IRDs with undefined clinical diagnosis. Participants underwent a complete ophthalmologic examination followed by genetic counseling. A custom AmpliSeq™ panel of 72 IRD-related genes was designed for the analysis and tested using Ion semiconductor Next-Generation Sequencing (NGS). Potential disease-causing mutations were identified in 59.1% of probands, comprising mutations in 16 genes. The highest diagnostic yields were achieved for BMD, LCA, USH, and STGD patients, whereas RP confirmed its high genetic heterogeneity. Causative mutations were identified in 17.6% of probands with undefined diagnosis. Revision of the initial diagnosis was performed for 9.6% of genetically diagnosed patients. This study demonstrates that NGS represents a comprehensive cost-effective approach for IRDs molecular diagnosis. The identification of the genetic alterations underlying the phenotype enabled the clinicians to achieve a more accurate diagnosis. The results emphasize the importance of molecular diagnosis coupled with clinic information to unravel the extensive phenotypic heterogeneity of these diseases.

## 1. Introduction

Inherited Retinal Dystrophies (IRDs) are a heterogeneous group of eye disorders characterized by rod and/or cone photoreceptor cells degeneration, which include Retinitis Pigmentosa (RP), Leber Congenital Amaurosis (LCA), Stargardt Disease (STGD), Best Macular Dystrophy (BMD), and syndromic forms such as Usher Syndrome (USH). The overall prevalence of these disorders is ~1 in 4,000 individuals for RP, ~1 in 90,000 individuals for LCA and USH, ~1 in 5,000–10,000 individuals for STGD, and 1/5000–1/67000 for BMD (http://www.orpha.net). Classification of IRDs considers the principal site of retinal dysfunction (rod, cone, retinal pigment epithelium, or inner retina), the mode of inheritance, the underlying gene defect, typical age of onset, rate of progression, and association with systemic syndromes. The genetic bases of IRDs are highly heterogeneous, with almost 150 genes currently known [RetNet, https://sph.uth.edu/retnet/] and a wide clinical and genetic overlap among the different disorders, with high phenotypic variability and genes associated with more than one phenotype. The inheritance of these diseases is also complex, with autosomal dominant (AD), autosomal recessive (AR), X-linked (XL), and even digenic patterns [1]. The extensive clinical and genetic heterogeneity in IRD, along with the variable age of onset, the incomplete penetrance, and unclear inheritance, hamper clinical diagnosis.

Recently, Next-Generation Sequencing (NGS) has been used for the genetic diagnosis of retinal diseases [2–6] and has been reported as a cost-effective approach [7, 8] with a wide range of reported mutation detection rates related to differences in number of genes analyzed, NGS platform, and cohort size but above all composition of the study case phenotypes. We therefore present a multidisciplinary approach coupled with a comprehensive NGS amplicon-based strategy to explore IRD genetic complexity and evaluate genotype-phenotype correlations.

## 2. Patients and Methods

This study was approved by the ethics committee (Comitato Etico di Modena, Modena, Italy). The procedures followed were in accordance with the Helsinki Declaration of 1975, as revised in 2000, and samples were obtained after patients had provided written informed consent.

A total of 109 samples were collected, including 88 IRDs affected probands with unknown molecular diagnosis and 21 healthy family members (Table 1). Subjects were recruited at the Medical Genetics Unit of the University Hospital of Modena (70 samples), at the Medical Genetics Unit of Parma University Hospital (15 samples) and Medical Genetics Unit of Policlinico Sant'Orsola Malpighi, Bologna (24 samples). All subjects underwent a complete ophthalmologic examination (visual acuity, anterior segment and fundus examination, spectral domain-optical coherence tomography, electroretinogram, and/or electrooculogram) followed by genetic counseling. When indicated fundus autofluorescence imaging and visual field were also performed. Clinical information for the patients with identified pathogenic mutations is shown in Supplementary Table  1 (in Supplementary Material available online at http://dx.doi.org/10.1155/2016/6341870). Clinical diagnoses of participating subjects included RP, USH (hearing impairment + RP), LCA, STGD, BMD, and IRDs not otherwise specified or with imprecisely defined clinical diagnosis. Four control patients with known molecular diagnosis were used to validate our method.

### 2.1. AmpliSeq Panel Design and Ion Torrent™ PGM™ Library Preparation and Sequencing

The Ion AmpliSeq technology (Life Technologies Ltd., Paisley, UK) was used to design a panel of 72 genes (Supplementary Table  2) associated with the following IRD forms: RP, LCA, STGD, BMD, and USH [RetNet, https://sph.uth.edu/retnet/]. The Ion AmpliSeq Designer tool (https://www.ampliseq.com/browse.action) generated an optimized primers design encompassing the coding DNA sequence of the selected genes, for a total of 1.649 amplicons divided into two pools to optimize coverage and multiplex PCR conditions. Libraries were prepared using the Ion AmpliSeq Library Kit 2.0 starting from 15 ng of gDNA/pool according to manufacturer's recommendations. Template preparation was performed using an Ion OneTouch™ 2 System following the latest version of the manufacturer's manuals. The template positive Ion Sphere Particles (ISPs+) were sequenced on an Ion Torrent Personal Genome Machine® (PGM) System (Life Technologies Ltd., Paisley, UK) using the Ion 318™ Chip kit v2 following the Ion PGM Sequencing 200 Kit v2 manual.

### 2.2. Sanger Sequencing

Sanger sequencing was performed to validate* CNGB1* c.875-5_891dup mutation (identified with an anomalous distribution of NGS reads attributable to amplification problems due to the insertion itself located at the end of the target region) and to sequence* RPGR *ORF15 partially uncovered by the NGS panel. Primers for PCR and sequencing are shown in Supplementary Table  3. The following conditions were used: a 50 *μ*L PCR reaction containing 100 ng of DNA, 100 pmol of forward and reverse primers, 5 *μ*L of buffer, and 0.5 *μ*L of Taq Expand High Fidelity™ DNA Polymerase (Roche). PCR amplification (see Supplementary Table  3) was performed using a Gene Amp PCR System 9700 (Applied Biosystems, California, USA). The resultant amplicons were purified using High Pure PCR Product Purification Kit (Roche). Additional primers for* RPGR* sequencing were used. The sequencing reactions were performed with BigDye Terminator v1.0 (Life Technologies) and run on ABI PRISM® 3130XL Genetic Analyzer (Life Technologies). Due to sequence composition and technical difficulties, part of* RPGR* ORF15 (~250 bp, chrX: 38145343–38145593) could not be accurately sequenced with Sanger sequencing.

### 2.3. Data Analysis

Samples were processed using the Ion Torrent Suite™ (TS) Software for raw data processing and sequence alignment to the human genome reference sequence hg19. The TS Variant Caller was used for the detection of germline variants that were subsequently analyzed using the following optimized filtering and annotation pipeline. Annovar [9] and Variant Effect Predictor (VEP) [10] were used to functionally annotate the detected variants, retrieving RefSeq gene annotation, dbSNP rs identifiers, ClinVar accession, and allele frequency observed in the population (1000-Genome Project, NHLBI GO Exome Sequencing Project ESP6500SI-V2, Exome and Aggregation Consortium ExAC 0.3). Variants with low coverage or low frequency (<30 reads or <30%, resp.) were filtered out. The synonymous variants and variants having an allele frequency greater than 1% reported in the population were discarded as well. In addition, an internal database, built with all variants present in our cohort of processed samples, allowed recognizing and classifying as polymorphisms variants not listed in public databases. Variants were further annotated with conservation scores and functional predictions listed in dbNSFP [11–13], a database which compiles scores from various prediction algorithms, among which are SIFT, Polyphen2, LRT, MutationTaster, MutationAssessor, and FATHMM. Retina International (http://www.retina-international.org/), RPGR database (http://rpgr.hgu.mrc.ac.uk/index.php?select_db=RPGR), CEP290base (http://cep290base.cmgg.be/), and BEST1 LOVD database (http://www-huge.uni-regensburg.de/BEST1_database) were used to explore additional annotations and literature information, if present. Splice-altering predictions were obtained using the online tools Human Splicing Finder (HSF 3.0) [14] and NNSPLICE 0.9 [15] and the databases dbscSNV [16] and SPIDEX [17], which provide predicted effects for all of the potential variants within splicing consensus regions or across the entire genome, respectively. For the prioritization of pathogenetic mutations, the evaluation of inheritance mode was taken into account, along with segregation information coming from the sequencing of healthy family members, if available.

NGS procedure and data analysis were tested on the four control samples with known molecular diagnosis as proof of concept. In all cases the previously identified variants were correctly detected and prioritized as pathogenic variants.

## 3. Results

**Figure 1:**
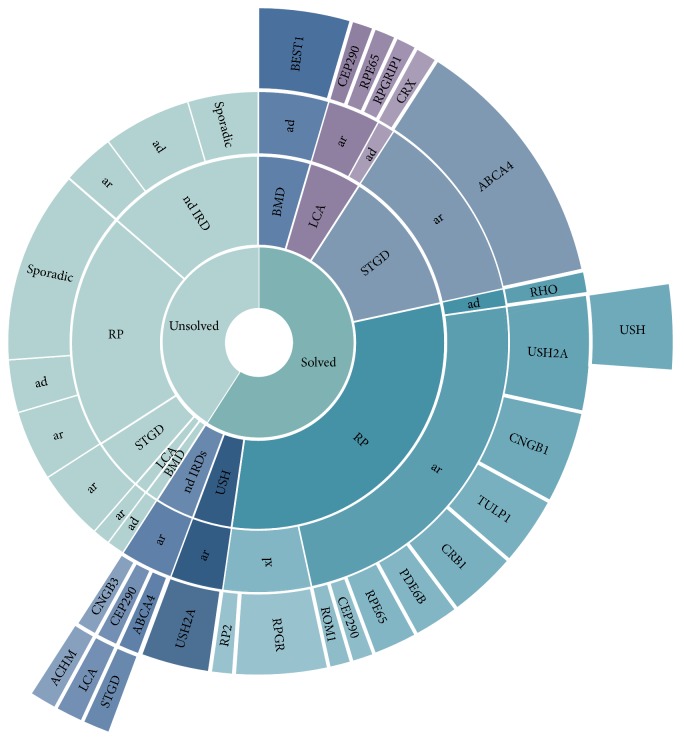
The chart summarizes the diagnostic yields obtained for the clinical subtypes of this study. The different levels of circles (from inner to outside) specify clinical diagnoses, inheritance mode, mutated genes, and clinical reassessment.

A cohort of 109 samples (Table 1), including 88 IRDs affected probands without molecular diagnosis and 21 unaffected family members, was analyzed by the newly developed system based on NGS and data analysis. A total of 19 sequencing runs were performed (6 samples/Ion Chip 318), obtaining on average a mean coverage of 450 mapped reads, with 92% mean uniformity and 97.6% (SD ± 1.4) of target regions covered at least 30x (96.2% > 50x). For each sample, 242 raw variants were detected on average. Annotation and filtering procedure resulted in the identification of possibly causative mutations in 59.1% of patients (*n* = 52/88) (Table 2, Figure 1). The majority of the obtained molecular diagnoses were consistent with the subject's clinical presentation and family history.

We found pathogenic mutations in 16 genes, with the most recurrent being* ABCA4* for STGD and* USH2A* for RP/USH patients. The majority of the mutated genes were inherited with an AR pattern (78.9%), followed in order by AD (11.5%) and XL (9.6%) inheritance. The majority of cases displaying recessive inheritance were compound heterozygous of two different pathogenic variants, in line with the low frequency of consanguineous marriages in Italy

Identified candidate pathogenic mutations are shown in Table 3. Overall, 63 different mutations were identified: 62.5% of variants were already reported in previous studies, while 37.5% were novel. Among the list of novel variants, 56% were missense predicted to have deleterious protein functional effect by the prediction algorithms described in the Patients and Methods (predicted to be damaging by at least three of the applied algorithms), and 44% were frameshift, nonsense, or splice-site mutations that might severely affect protein function. Notably, 12% of identified variants were located within splicing consensus regions, and additional 12% were exonic variants predicted to alter splicing through enhancer/silencer motif modification or the creation of new potential donor/acceptor sites.

Table 2 summarizes the mutation detection rates obtained for the different clinical subtypes of our study cohort. The highest diagnostic yields were achieved for BMD, LCA, USH, and STGD patients with well-defined clinical diagnosis, where the number of known genes associated with each disease is relatively limited.

For BMD cases, all diagnosed patients were heterozygous for mutations on* BEST1*. Three patients (mother and son) were found to harbour a novel* BEST1* missense mutation c.80G>C (p.Ser27Thr) located in the immediate N-terminus, in one of the four mutational hotspots regions in the highly conserved N-terminal half of the protein [18] and predicted to be deleterious by all interrogated algorithms.

For STGD patients, genetic diagnosis was achieved in 11 out of 14 (78.5% of the cases). All diagnosed patients in our cohort carried mutations on* ABCA4*. In 75% of the unsolved cases at least one* ABCA4* pathogenic allele was identified, suggesting the presence of disease-causing mutations lying outside the coding sequence covered by our panel, as reported in a previous study [19].

In LCA patients, causative mutations were identified in* CEP290, RPE65, RPGRIP1,* and* CRX* genes, and only one case remained unsolved (20% of the total LCA cases), whereas all Usher 2 syndrome cases were found to carry mutations in USH2A gene.

For RP patients, genetic diagnosis was achieved in 27 out of 45 (60% of the cases), involving mutations in 11 different genes: confirming that these phenotypes are genetically heterogeneous (Figure 1). Dominant mutations were identified in* RHO* gene, whereas* USH2A, CNGB1,* and* TULP1* were the most recurrently mutated genes in ARRP. X-linked inheritance was established for 5 RP male patients (4 probands had mutations in* RPGR*, whereas one had a mutation in RP2). The identification of* USH2A* as the defective gene in patients with initial clinical diagnosis of RP was followed by audiometric testing to establish if there were any hearing deficiencies. A hearing impairment was found in 2 cases out of 5 leading to clinical reassessment and final diagnosis of USH (Table 2).

For patients with IRD without a defined clinical diagnosis or with unclear disease manifestations, we identified causative mutations in 7 out of 17 probands (23.5% of the total IRD cases). In two cases the molecular results allowed a refined clinical diagnosis: a compound heterozygosity of two mutations in* CEP290* led to a genetic diagnosis of LCA in a patient with initial diagnosis of North Carolina or Stargardt macular dystrophy, whereas a homozygous pathogenic variant in* ABCA4* was found in a patient with tapetoretinal degeneration.

In 36 patients (12 familiar and 24 sporadic) the molecular analysis did not achieve any definitive result, even after the analysis of the healthy family members, which was performed in 8 cases. Half of the cases with a negative test result (18 out of 36) were affected by RP. The additional analysis of the* RPGR* ORF15 (a mutational hotspot which was nonsufficiently covered in our panel) for the male patients with a sporadic or suspected X-linked pattern of inheritance (10 patients) by Sanger sequencing yielded no additional mutations.

## 4. Discussion

The results of the present study confirm that high-throughput Next-Generation Sequencing represents a comprehensive cost-effective approach for the molecular diagnosis of Inherited Retinal Dystrophies (IRDs), achieving a molecular diagnosis for 59.1% of the studied cases. More specifically, among the different clinical phenotypes, the highest detection rates were achieved for BMD, LCA, USH, and STGD patients, in whom the genetic test clearly confirmed the clinical diagnoses (Table 2). The results of the RP and of the not defined IRD cohorts, instead, demonstrated the high genetic heterogeneity of this diseases and the essential contribution of our NGS analysis to achieving an accurate diagnosis, with the involvement of 12 different genes in 28 sporadic cases. Revision of the initial diagnosis, performed for 9.6% of the genetically diagnosed patients, further emphasizes the importance of a comprehensive genotype/phenotype analysis to unravel the extensive heterogeneity of these diseases. Notably, a remarkable fraction of identified variants are splice-altering mutations (25% of the total mutation burden, 16 out of 64), located within splicing consensus regions, or exonic variants predicted to cause enhancer/silencer motif modification or the creation of new potential donor/acceptor, which are amenable to the antisense-mediated splicing-correction approaches, as recently reported for several genetic diseases, including* CEP290*-caused LCA [20, 21].

The prevalence of IRD and most importantly the frequency of gene mutations causing those diseases are not well characterized in Italy and only few data have been reported [22–24].* RPE65, CRB1,* and* GUCY2D* were identified as the most prevalent mutated genes in Italian LCA patients [22] and* RHO* was reported to be the gene most commonly responsible for ADRP [23] and* EYS* the most recurrent for nonsyndromic ARRP and sporadic cases [24]. Our study contributes only partially to the knowledge of the gene mutation frequencies, since each IRD type is represented by small cohorts of cases (i.e., the LCA and dominant RP phenotypes were accounted for by 5 and 6 cases, resp.), and some probands of other ethnicities have been included too. Indeed, regarding LCA, we identified mutations in CEP290,* RPE65*,* CRX*, and* RPGRIP1* genes.

For ADRP,* RHO* was identified to be responsible for the phenotype in one case, whereas, in ARRP and sporadic RP,* USH2A, CNGB1,* and* TULP1* were the most recurrently mutated genes.* RPE65* mutations were found in two ARRP cases: in one more case, still unsolved, a single* RPE65* heterozygous pathogenic variant was found.* ROM1* compound heterozygosity was established in one RP proband, suggesting a mechanism of recessive inheritance for this gene associated with dominant and digenic forms. X-linked inheritance was established for 5 RP affected probands, with* RPGR* and* RP2* identified as the disease-causing gene in 4 cases and 1 case, respectively. All BMD diagnosed patients were heterozygous for mutations on* BEST1* gene, the major gene responsible for Best's juvenile form [25], whereas the 78.5% of patients with clinically diagnosed STGD carried pathogenic variants on* ABCA4 *[26].

Similarly to a recent study [6], the clinical sensitivity of our NGS analysis was not uniform, with the highest diagnostic yields obtained in conditions where the disease-causing genes have been nearly all identified.

Direct comparison of our findings with other recently published NGS studies [2–6, 27] is not straightforward, due to differences in the number of genes analyzed but especially due to composition and relative representation of the different phenotypes in the patients cohorts. However, the finding of* USH2A* and* ABCA4* as the most mutated genes for RP/USH and STGD patients is consistent with previous reports [27–29]. In our RP cohort,* USH2A* is followed by* CNGB1* and* RPGR*. These two genes, already reported among the most frequently mutated genes in IRD patients [29], were not highly frequently altered in the Saudi population [6] or in a large cohort of Western European and South Asian individuals [27]. Also, we did not find any alteration in* EYS*, one of the top three genes contributing to IRD in other populations [28, 29].

The different gene alterations identified in our LCA cohort (*CEP290, RPE65, RPGRIP1,* and* CRX* genes) were consistent with the different disease manifestations of the analyzed patients, in accordance with the specific clinical features described for each of the LCA-associated genes [30, 31]. Less direct is the correlation between the genes involved and the phenotypic features in RP, due to the known contribution of environmental factors to late-childhood- and adult-onset-diseases.

Allelic heterogeneity, with different mutations in the same gene causing different phenotypes, is evident also in* USH2A*-related retinal disease. Genotype-phenotype correlations observed in our cohort were in accordance with the allelic hierarchy proposed in a recent study [32], supporting the model that USH represents the null phenotype consequent upon severe* USH2A* defects, whereas milder mutations in at least one allele result in a pure retinal phenotype associated with normal auditory function.

IRD genetic heterogeneity, reflected in the identification of mutations in many genes with a considerable number of previously undescribed alterations, supported the conclusion that molecular diagnosis of these disorders should rely on massive parallel multigene sequencing. Nevertheless, for 36 probands, including 12 familiar cases and 24 unrelated probands, our NGS procedure did not result in the identification of a clear genetic cause of the disease. Some subjects may have mutations that cannot be detected by our amplicon-based approach, such as deep intronic mutations, copy-number variations, or large deletions. In the perspective of the design of a more complete new version of the panel, additional deep intronic regions reported in the literature as carrying disease-causing mutations [19, 33, 34] or a higher exon padding (5 bp in our design, up to 100 bp available in the current pipeline version of the Ion AmpliSeq Designer tool) could be implemented. Moreover, technical limitations, including the difficult amplification of* RPGR* ORF15, a mutational hotspot for X-linked RP, may have accounted for some of the missed diagnosis (our panel is presently covering only 30% of this critical exon), but the addition of the specific analysis by Sanger sequencing of the ORF15 of the* RPGR* gene in 10 males patients, with sporadic/X-linked RP and previously testing negative for pathogenic mutations using our NGS panel, did not reveal any mutation in the analyzed region. Finally, as an improvement to further support the pathogenicity of novel mutations identified in probands, the analysis of both affected and unaffected family member should be performed, when possible.

In some of the patients who tested negative we however identified single potentially pathogenic heterozygous mutations in recessive genes or novel heterozygous missense variants in dominant genes with unknown significance, lacking the appropriate level of evidence to classify them as disease-causing or not in concordance with patients' clinical presentations or family data. The contribution of these variants in combination with deep intronic mutations or large deletions is suspected but could not be demonstrated with the present technique.

Database incompleteness further complicates variant interpretation. Two probands with BMD phenotype and* BEST1* mutation were found to harbour also heterozygous mutation in* RHO* (c.578C>T, p.Thr193Met), which was predicted to be damaging and listed as associated with ADRP in a public database [http://www.retina-international.org/sci-news/databases/mutation-database] but in our cohort was carried also by healthy subject, reinforcing the need of a critical interpretation of the molecular findings in view of the phenotypic features of the patients with IRD until a more thorough knowledge of the frequency of the variants and a critical amount of data present in the public disease databases are reached.

In conclusion, by presenting profoundly different mutation rates varying according to the clinical diagnosis and by reporting 9.61% of cases of reassessment of the initial diagnosis on the basis of the results of the test, our study reinforces the need of a multidisciplinary work-up before and after the genetic testing, due to the implications of the results in terms of risk assessment for family members and inclusion in gene-based clinical trials.

## Supplementary Material

Clinical information for the patients with identified pathogenic mutations is reported in Supplementary Table1. Supplementary Table2 lists the genes included in the designed NGS panel and their associated retinal disease. Primers used for PCR and Sanger sequencing are reported in Supplementary Table 3.

## Figures and Tables

**Table 1:** Patients cohort.

Clinical diagnosis	Number of cases	Healthy relatives	Familiar Cases (number of families)	Presumed inheritance in family				Sex		Age at genetic counseling	
				Sporadic	AD	AR	XL	M	F	Range	Median
BMD	4		2 (1)		4			1	3	12–65	58
LCA	5	5		1		4		2	3	5–85	9
STGD	14		6 (3)			14		5	9	8–59	28
RP	45	12	9 (4)	14	6	20	5	25	20	2–73	47.5
USH	3					3		2	1	33–53	51
nd IRD	17	4	6 (2)	6	6	5		13	4	2–62	35
Total	88	21	23 (10)	21	16	46	5	48	40	2–85	37

BMD: Best Macular Dystrophy; LCA: Leber Congenital Amaurosis; STGD: Stargardt disease; RP: Retinitis Pigmentosa; USH: Usher syndrome; nd IRD: inherited retinal degeneration not otherwise specified without precisely defined diagnosis; AD: autosomal dominant; AR: autosomal recessive; XL: X-linked; M: male; F: female.

**Table 2:** Diagnostic yields for the clinical subtypes of this study.

Clinical diagnosis	Cases (*n*)	Genetic diagnosis (*n*)	Unsolved cases (*n*)	Clinical reassessment (final diagnosis)	Diagnostic yield (%)
BMD	4	4	—		100
LCA	5	4	1		80
STGD	14	11	3		78.5
RP	45	27	18	2 (USH)	60.0
USH	3	3	—		100
nd IRD	17	3	14	3 (ACHM, LCA, STGD)	17.6
Total	88	52	36	5	59.1

BMD: Best Macular Dystrophy; LCA: Leber Congenital Amaurosis; STGD: Stargardt Disease; RP: Retinitis Pigmentosa; USH: Usher Syndrome; nd IRD: inherited retinal degeneration not otherwise specified without precisely defined diagnosis; ACHM: Achromatopsia.

**Table 3:** 

Patient ID	Family	Clinical diagnosis	Clinical reassessment	Genotype	Inheritance	Gene	Mutation type	Region	cds change

IRD027		STGD		Comp Het	ar	*ABCA4*	Splice_region	INTRON_40	c.5714+5G>A
						*ABCA4*	Frameshift	EXON_11	c.1375delA
IRD036	Familiar case	STGD		Comp Het	ar	*ABCA4*	Stop_gained	EXON_14	c.2099G>A
						*ABCA4*	Splice_region syn	EXON_6	c.768G>T
IRD037		STGD		Comp Het	ar	*ABCA4*	Stop_gained	EXON_14	c.2099G>A
						*ABCA4*	Splice_region syn	EXON_6	c.768G>T
IRD042	Familiar case	STGD		Comp Het	ar	*ABCA4*	Missense	EXON_42	c.5882G>A
						*ABCA4*	Missense	EXON_6	c.634C>T
IRD043		STGD		Comp Het	ar	*ABCA4*	Missense	EXON_42	c.5882G>A
						*ABCA4*	Missense	EXON_12	c.1622T>C
IRD050		STGD		Comp Het	ar	*ABCA4*	Missense	EXON_16	c.2461T>A
						*ABCA4*	Missense	EXON_15	c.2300T>A
IRD054		STGD		Comp Het	ar	*ABCA4*	Stop_gained	EXON_47	c.6445C>T
						*ABCA4*	Missense	EXON_42	c.5882G>A
IRD055		STGD		Comp Het	ar	*ABCA4*	Missense	EXON_19	c.2842C>T
						*ABCA4*	Missense	EXON_15	c.2300T>A
IRD061		STGD		Comp Het	ar	*ABCA4*	Missense	EXON_42	c.5882G>A
						*ABCA4*	Missense	EXON_28	c.4139C>T
IRD062		STGD		Comp Het	ar	*ABCA4*	Missense	EXON_42	c.5882G>A
						*ABCA4*	Missense	EXON_16	c.2549A>G
IRD073		nd IRD	STGD	Hom	ar	*ABCA4*	Missense	EXON_19	c.2894A>G
IRD077		STGD		Comp Het	ar	*ABCA4*	Missense	EXON_37	c.5285C>A
						*ABCA4*	Missense	EXON_15	c.2300T>A
IRD047		BMD		Het	ad	*BEST1*	Missense	EXON_2	c.73C>T
IRD057	Familiar case	BMD		Het	ad	*BEST1*	Missense	EXON_2	c.80G>C
IRD058		BMD		Het	ad	*BEST1*	Missense	EXON_2	c.80G>C
IRD064		BMD		Het	ad	*BEST1*	Missense	EXON_2	c.80G>C
IRD010		LCA		Comp Het	ar	*CEP290*	Missense	EXON_33	c.4237G>C
						*CEP290*	Frameshift	EXON_23	c.2390delA
IRD066		RP		Comp Het	ar	*CEP290*	Stop_gained	EXON_48	c.6640A>T
						*CEP290*	Frameshift	EXON_14	c.1219_1220delAT
IRD072		nd IRD	LCA	Comp Het	ar	*CEP290*	Missense	EXON_14	c.1298A>G
						*CEP290*	Frameshift	EXON_3	c.164_167delCTCA
IRD039		RP		Hom	ar	*CNGB1*	Frameshift	EXON_13	c.875-5_891dup
IRD052		RP		Comp Het	ar	*CNGB1*	Missense	EXON_29	c.2957A>T
						*CNGB1*	Frameshift	EXON_13	c.875-5_891dup
IRD068		RP		Comp Het	ar	*CNGB1*	Splicing, syn	EXON_26	c.2526C>T
						*CNGB1*	Missense	EXON_21	c.2153G>C
IRD085		RP		Hom	ar	*CNGB1*	Missense	EXON_23	c.2284C>T
IRD032		nd IRD	ACHM	Comp Het	ar	*CNGB3*	Splice_donor	INTRON_13	c.1578+1G>A
						*CNGB3*	Frameshift	EXON_10	c.1148delC
IRD029	Familiar case	RP		Hom	ar	*CRB1*	Missense	EXON_5	c.2200G>A
IRD030		RP		Hom	ar	*CRB1*	Missense	EXON_5	c.2200G>A
IRD031		RP		Hom	ar	*CRB1*	Missense	EXON_5	c.2200G>A
IRD035		LCA		Het	ad	*CRX*	Frameshift	EXON_4	c.514delC
IRD008		RP		Hom	ar	*PDE6B*	Splice_region	EXON_18	c.2193+1delG
IRD013		RP		Comp Het	ar	*PDE6B*	Missense	EXON_4	c.794G>A
					ar	*PDE6B*	Intron	INTRON_8	c.1108-10G>A
IRD026		RP		Het	ad	*RHO*	Missense	EXON_3	c.568G>T
IRD016		RP		Comp Het	ar	*ROM1*	Missense	EXON_1	c.178C>A
						*ROM1*	Missense	EXON_1	c.323C>T
IRD033		RP		Hem	xl	*RP2*	Frameshift	EXON_2	c.382_383delTT
IRD076		RP		Hom	ar	*RPE65*	Missense	EXON_2	c.65T>C
IRD001		RP		Comp Het	ar	*RPE65*	Missense	EXON_2	c.65T>C
						*RPE65*	Frameshift	EXON_9	c.893delA
IRD074		LCA		Hom	ar	*RPE65*	Missense	EXON_5	c.430T>G
IRD002		LCA		Comp Het	ar	*RPGRIP1*	Frameshift	EXON_15	c.2225_2226delGA
						*RPGRIP1*	Frameshift	EXON_17	c.2795_2796insT
IRD012		RP		Hem	xl	*RPGR*	Missense	EXON_8	c.785C>G
IRD067		RP		Hem	xl	*RPGR*	Missense	EXON_8	c.814G>T
IRD075		RP		Hem	xl	*RPGR*	Missense, Splice_region	EXON_2	c.154G>A
IRD017		RP		Hem	De novo	*RPGR*	Frameshift	EXON_2	c.89delT
IRD059	Familiar case	RP		Comp Het	ar	*TULP1*	Missense	EXON_15	c.1590C>G
						*TULP1*	Missense	EXON_13	c.1255C>T
IRD060		RP		Comp Het	ar	*TULP1*	Missense	EXON_15	c.1590C>G
						*TULP1*	Missense	EXON_13	c.1255C>T
IRD041		RP		Comp Het	ar	*TULP1*	Splice_region	INTRON_14	c.1496-6C>A
						*TULP1*	Missense	EXON_14	c.1445G>A
IRD007		USH		Comp Het	ar	*USH2A*	Missense	EXON_63	c.12420T>G
						*USH2A*	splice_region, syn	EXON_28	c.5775A>T
IRD009		USH		Comp Het	ar	*USH2A*	Missense	EXON_63	c.13546G>T
						*USH2A*	splice_region, Missense	EXON_10	c.1645T>C
IRD021		RP		Comp Het	ar	*USH2A*	Missense	EXON_69	c.14995A>G
						*USH2A*	Missense	EXON_8	c.1481A>G
IRD023	Familiar case	RP	USH	Comp Het	ar	*USH2A*	Missense	EXON_13	c.2296T>C
						*USH2A*	Frameshift	EXON_3	c.545_548delAAGA
IRD024		RP	USH	Comp Het	ar	*USH2A*	Missense	EXON_13	c.2296T>C
						*USH2A*	Frameshift	EXON_3	c.545_548delAAGA
IRD038		RP		Comp Het	ar	*USH2A*	Missense	EXON_13	c.2296T>C
						*USH2A*	Missense	EXON_13	c.2276G>T
IRD084		USH		Hom	ar	*USH2A*	Frameshift	EXON_69	c.14977_14978delTT
IRD034		RP		Hom	ar	*USH2A*	Missense	EXON_63	c.12574C>T

Patient ID	Protein change	Frequency (%)	Coverage (# reads)	Segregation and unaffected siblings	Functional predictions (dbNSFP)	Splicing predictions			Reference
						Human Splicing Finder	dbscSNV	SPIDEX	

IRD027		44.9	514			Broken WT Donor Site	0.999|0.988	−3.21	PMID: 15494742
	p.Thr459GlnfsX2	47.7	1179						PMID: 21911583
IRD036	p.Trp700X	48.2	303		.|..|N|A|.|.|.|.|.|D				PMID: 11702214
	p.Val256Val	47.2	53			Broken WT Donor Site	1.000|0.952	−2.43	PMID: 12037008
IRD037	p.Trp700X	44.5	110		.|..|N|A|.|.|.|.|.|D	New Acceptor Site		−5.41	PMID: 11702214
	p.Val256Val	48.3	29			Broken WT Donor Site	1.000|0.952	−2.43	PMID: 12037008
IRD042	p.Gly1961Glu	47.1	1325		D|DD|D|D|N|D|D|D|D|D				PMID: 9295268
	p.Arg212Cys	49.1	432		D|DD|D|A|M|D|D|D|D|D				PMID: 11726554
IRD043	p.Gly1961Glu	46.9	796		D|DD|D|D|N|D|D|D|D|D				PMID: 9295268
	p.Leu541Pro	51.9	727		D|DD|D|A|M|D|D|D|D|D				PMID: 11527935
IRD050	p.Trp821Arg	43.8	309		D|DD|D|D|H|T|D|D|D|D				PMID: 11527935
	p.Val767Asp	46.3	452		D|BB|D|D|M|D|T|D|D|D				PMID: 15494742
IRD054	p.Arg2149X	49.1	422		.|..|D|A|.|.|.|.|.|D	New ESS site		−58.3	PMID: 12202497
	p.Gly1961Glu	49.4	1448		D|DD|D|D|N|D|D|D|D|D				PMID: 9295268
IRD055	p.Arg948Cys	52.0	175		T|BB|N|D|L|D|T|T|N|N				This study
	p.Val767Asp	51.5	437		D|BB|D|D|M|D|T|D|D|D				PMID: 15494742
IRD061	p.Gly1961Glu	50.0	729		D|DD|D|D|N|D|D|D|D|D				PMID: 9295268
	p.Pro1380Leu	55.8	437		D|DP|N|A|M|D|D|D|D|D	New ESS site		−5.44	PMID: 11726554
IRD062	p.Gly1961Glu	100	787		D|DD|D|D|N|D|D|D|D|D				PMID: 9295268
	p.Tyr850Cys	49.4	176		D|DD|D|D|M|T|D|D|D|D				PMID: 23096905
IRD073	p.Asn965Ser	100	225		D|DD|D|D|L|D|D|D|D|D				PMID: 9054934
IRD077	p.Ala1762Asp	50.8	259		D|DD|D|A|M|D|D|D|D|D				PMID: 15192030
	p.Val767Asp	51.4	752		D|BB|D|D|M|D|T|D|D|D				PMID: 15494742
IRD047	p.Arg25Trp	56.0	348		D|DD|U|D|M|D|D|D|D|D	New Donor Site, New ESS site			PMID: 10798642
IRD057	p.Ser27Thr	46.8	344		D|DD|U|D|H|D|D|D|D|D				This study
IRD058	p.Ser27Thr	45.5	317		D|DD|U|D|H|D|D|D|D|D				This study
IRD064	p.Ser27Thr	47.1	453		D|DD|U|D|H|D|D|D|D|D				This study
IRD010	p.Asp1413His	49.2	413		D|BB|D|D|N|T|T|T|N|D				ClinVar: RCV000082249.5
	p.Lys797SerfsX2	30.1	163						This study
IRD066	p.Lys2214X	47.5	705		.|..|D|A|.|.|.|.|.|D	ESE Site Broken		−86.6	This study
	p.Met407GlufsX14	51.1	225						PMID: 17724218
IRD072	p.Asp433Gly	53.4	116		T|DP|D|D|L|T|T|T|D|D	New ESS site, New donor site			This study
	p.Thr55SerfsX3	43.2	243						PMID: 20690115
IRD039	p.Gly298CysfsX13	100^*∗*^							This study
IRD052	p.Asn986Ile	51.7	471		D|DD|D|D|M|D|D|D|D|D				PMID: 21147909
	p.Gly298CysfsX13	26,7^*∗*^	258						This study
IRD068	Thr842Thr	52.1	431			ESE Site Broken			This study
	p.Gly718Ala	47.1	153		D|PP|D|D|M|T|T|T|D|D				This study
IRD085	p.Arg762Cys	100	57		D|DD|D|D|H|D|D|D|D|D				This study
IRD032		47.8	907			Broken WT Donor Site		−8.56	PMID: 15657609
	p.Thr383IlefsX13	46.5	588						PMID: 15657609
IRD029	p.Gly734Arg	100	397		D|DD|.|D|M|T|D|D|D|D				This study
IRD030	p.Gly734Arg	100	397		D|DD|.|D|M|T|D|D|D|D				This study
IRD031	p.Gly734Arg	100	397		D|DD|.|D|M|T|D|D|D|D				This study
IRD035	p.Pro172LeufsX15	50.5	521						This study
IRD008		100	395	Brother: Het		Broken WT Donor Site			This study
IRD013	p.Arg265Gln	51.7	319	n.a.	T|DD|D|D|L|T|T|T|N|D				ClinVar: RCV000178068.1
		54.7	75	Mother: Het			0.001|0.096		PMID: 8698075
IRD026	p.Asp190Tyr	44.6	168		D|DD|D|D|M|T|T|T|D|D				PMID: 8401533
IRD016	p.Pro60Thr	56.1	278		T|BB|N|N|L|T|T|T|N|N				PMID: 8595413
	p.Thr108Met	52.8	108		T|PB|N|D|L|T|T|T|N|D				PMID: 8595413
IRD033	p.Leu129ValfsX9	100	392						This study
IRD076	p.Leu22Pro	100	495		T|BB|D|D|M|D|D|D|N|D				PMID: 9801879
IRD001	p.Leu22Pro	46.3	257	Brother: wt	T|BB|D|D|M|D|D|D|N|D				PMID: 9801879
	p.Lys298SerfsX27	98	150	Brother: wt					PMID: 11462243
IRD074	p.Tyr144Asp	100	430	Father: Het	D|DD|D|D|M|D|D|D|D|D				PMID: 11462243
IRD002	p.Glu743ArgfsX24	48.8	570	Father: Het					This study
	p.Glu933X	48.8	400	Mother: Het					This study
IRD012	p.Ala262Gly	100	280		T|BB|N|N|L|D|T|T|N|N				This study
IRD067	p.Gly272Cys	100	155		D|DD|D|D|H|D|D|D|D|D				This study
IRD075	p.Gly52Arg	100	348		D|DP|U|D|M|T|T|T|D|D	Broken WT Donor Site			PMID: 15364249
IRD017	p.Phe30SerfsX38	100	113	Brother: wt Female twin: wt					This study
IRD059	p.Ile530Met	50.6	682		D|DD|D|D|H|D|D|D|D|N				This study
	p.Arg419Trp	49.5	645		D|DD|D|D|H|D|D|D|D|D				PMID: 25342620
IRD060	p.Ile530Met	51.0	655		D|DD|D|D|H|D|D|D|D|N				This study
	p.Arg419Trp	45.3	575		D|DD|D|D|H|D|D|D|D|D				PMID: 25342620
IRD041		54.1	727	Father: Het			0.005|0.419		PMID: 9660588
	p.Arg482Gln	48.5	485	Mother: Het	D|DD|D|D|H|D|D|D|D|D	New Acceptor Site		−1.28	PMID: 22665969
IRD007	p.Cys4140Trp	50.5	214		D|DD|D|D|M|T|T|T|D|D				This study
		49.5	398			Broken WT Donor Site	0.998|0.986	−4.24	This study
IRD009	p.Gly4516Trp	53.8	239		D|DD|U|D|H|T|D|D|D|D				This study
	p.Cys549Arg	49.2	566		D|DD|U|D|H|D|D|D|D|D		0.417|0.520		This study
IRD021	p.Thr4999Ala	51.0	400		D|DD|U|D|M|T|T|T|D|D				This study
	p.Tyr494Cys	49.0	400		D|DD|N|D|L|T|T|T|D|D				This study
IRD023	p.Cys766Arg	39.0	82		D|DD|D|D|H|D|D|D|D|D				PMID: 23591405
	p.Lys182ArgfsX9	61.4	202						This study
IRD024	p.Cys766Arg	43.5	124		D|DD|D|D|H|D|D|D|D|D				PMID: 23591405
	p.Lys182ArgfsX9	48.0	225						This study
IRD038	p.Cys766Arg	47.2	89		D|DD|D|D|H|D|D|D|D|D				PMID: 23591405
	p.Cys759Phe	51.1	90		D|DD|D|A|H|D|D|D|D|D				PMID: 10775529
IRD084	p.Phe4993ProfsX7	100	483						PMID: 24944099
IRD034	p.Arg4192Cys	100	515		D|DP|N|D|M|D|D|D|D|D				PMID: 24498627

ACHM: Achromatopsia; ad: autosomal dominant; ar: autosomal recessive; BMD: best macular disease; Comp Het: compound heterozygous; ESE: exonic splicing enhancer; ESS: exonic splicing silencer; Hem: Hemizygous; Het: heterozygous; Hom: homozygous; LCA: Leber Congenital Amaurosis; nd IRD: inherited retinal degeneration not otherwise specified without precisely defined diagnosis; RP: Retinitis Pigmentosa; STGD: Stargardt Disease; USH: Usher Syndrome; wt: wild-type; xl: X-linked. For nonsynonymous variants, predictions from dbNSFP are reported, comprising scores from the following alghoritms: SIFT | Polyphen2HDIV Polypehn2HVAR | LRT | MutationTaster | MutationAssessor | FATHMM | MetaSVM | MetaLR | PROVEAN | fathmm-MKL. For splicing variants, predictions from Human Splicing Finder, dbscSNV (ada_score|rf_score) and SPIDEX are reported. For SPIDEX, max dPSI is shown if lower than −1 (maximum mutation − induced change in the percentage of transcripts with the exon spliced in). Familiar case: the patients were from the same family. ^*^Sanger sequencing was performed to confirm mutation frequency.

## Abbreviations/Acronyms

AD: Autosomal dominant
AR: Autosomal recessive
BMD: Best Macular Dystrophy
IRDs: Inherited Retinal Dystrophies
LCA: Leber Congenital Amaurosis
NGS: Next-Generation Sequencing
RP: Retinitis Pigmentosa
STGD: Stargardt Disease
USH: Usher Syndrome
XL: X-linked.
