# Supplementary material for: Unravelling the Complexity of Inherited Retinal Dystrophies Molecular Testing: Added Value of Targeted Next-Generation Sequencing
Source: Biomed Res Int. 2016 Dec 29;2016:6341870. doi: 10.1155/2016/6341870 (PMC5227126; doi:10.1155/2016/6341870)
Supplement: Supplementary file 1 — Clinical information for the patients with identified pathogenic mutations is reported in Supplementary Table1. Supplementary Table2 lists the genes included in the designed NGS panel and their associated retinal disease. Primers used for PCR and Sanger sequencing are reported in Supplementary Table 3. [file 6341870.f1.pdf]

Supplementary Table 1

Sample ID	Clinical diagnosis	Family	Consanquinity in the family	Etnicity	Gender	Age at diagnosis	Hearing Loss	Visual Acuity RE	Visual Acuity LE	Fundus changes	OCT	ERG	EOG	Color vision	Presenting symptoms	Visual Field	Genetic diagnosis Gene	Genetic diagnosis Genotype	Genetic diagnosis Inheritance	Clinical reassessment
IRD027	STGD		no	European	F	45	no	5/10	7/10	MA (BE)	AP,AR (BE)	normal (BE)	abnormal (BE)	na	CVL	central scotoma	*ABCA4*	Comp Het	ar	
IRD036	STGD	familiar case	no	European	F	34	no	LP	LP	MA,RA,PIG (BE)	AP,AR,AM (BE)	extinct (BE)	abnormal (BE)	na	VI	central scotoma+peripheral deficit	*ABCA4*	Comp Het	ar	
IRD037	STGD		no	European	M	27	no	HM	HM	MA,RA,PIG (BE)	AP,AR,AM (BE)	extinct (BE)	abnormal (BE)	na	VI	central scotoma+peripheral deficit	*ABCA4*	Comp Het	ar	
IRD042	STGD	familiar case	no	European	F	34	no	4/10	1/10	MA (BE)	AP,AR (BE)	normal (BE)	abnormal (BE)	na	CVL	central scotoma	*ABCA4*	Comp Het	ar	
IRD043	STGD		no	European	F	13	no	7/10	8/10	MA (BE)	AP,AR (BE)	normal (BE)	abnormal (BE)	na	CVL	central scotoma	*ABCA4*	Comp Het	ar	
IRD050	STGD		no	European	F	45	no	1/10	1/10	MA (BE)	AP,AR (BE)	normal (BE)	abnormal (BE)	na	CVL	central scotoma	*ABCA4*	Comp Het	ar	
IRD054	STGD		no	European	M	21	no	20/125	20/125	MA (BE)	AP,AR (BE)	na	na	na	CVL	na	*ABCA4*	Comp Het	ar	
IRD055	STGD		no	European	M	59	no	20/125	20/80	FF, extrafoveal MA (BE)	extrafoveal AP,AR, intraretinal hyperereflective deposits (BE)	normal (BE)	na	na	CVL	na	*ABCA4*	Comp Het	ar	
IRD061	STGD		no	European	F	30	no	20/125	20/125	MA, FF (BE)	AP,AR (BE)	normal (BE)	Abnormal; Indice di Arden 1,8 (RE) 1,9 (LE)	normal	CVL	central scotoma	*ABCA4*	Comp Het	ar	
IRD062	STGD		no	European	F	29	no	20/63	20/25	MA (BE)	AP,AR (BE)	na	na	normal	CVL	na	*ABCA4*	Comp Het	ar	
IRD073	nd IRD		yes	East Asian	M	47	no	HM	HM	MA, PIG (BE)	AP (BE)	na	na	na	CVL	na	*ABCA4*	Hom	ar	STGD
IRD077	STGD		no	European	M	8	no	20/200	20/160	MA (BE)	AP,AR (BE)	normal (BE)	normal (BE)	na	CVL	central scotoma	*ABCA4*	Comp Het	ar	
IRD047	BMD		no	European	F	63	no	CF	6/10	MA (RE); PIG (LE)	AP,AR (RE); hypereflective subretinal material (LE)	normal (BE)	abnormal (BE)	na	CVL	NA	*BEST1*	Het	ad	
IRD057	BMD	familiar case	no	European	F	53	no	20/80	20/50	MA (BE)	AP,AR (BE)	na	na	na	CVL	na	*BEST1*	Het	ad	
IRD058	BMD		no	European	M	12	no	20/32	20/25	vitelliform cyst (BE)	hypereflective subretinal material (BE)	na	na	na	CVL	na	*BEST1*	Het	ad	
IRD064	BMD		no	European	F	65	no	20/50	20/32	vitelliform lesion “scrambled egg”, extrafoveal yellow dots (BE)	subfoveal hyporeflectivity (BE)	na	na	na	CVL	na	*BEST1*	Het	ad	
IRD010	LCA		no	European	F	85	no	LP	LP	MA,RA,PIG (BE)	AP,AR,AM (BE)	extinct (BE)	abnormal (BE)	na	VI	complete loss	*CEP290*	Comp Het	ar	
IRD066	RP		no	European	M	23	no	10/10	10/10	AV, BS, PIG (BE)	extrafoveal AP (BE)	flat C (BE)	na	na	NYC	tubular	*CEP290*	Comp Het	ar	
IRD072	nd IRD		no	European	F	5	no	2/10	2/10	AM surrounded by pigmentary ring (BE)	AP in the macular region (BE)	reduced C (RE), mild reduced C (LE)	na	na	NYS	na	*CEP290*	Comp Het	ar	LCA
IRD039	RP		no	European	F	49	no	10/10	10/10	BS (BE)	extrafoveal AP (BE)	hypovolted (BE)	na	na	NYC	tubular	*CNGB1*	Hom	ar	
IRD052	RP		no	European	F	59	no	9/10	7/10	BS (BE)	extrafoveal AP (BE)	na	na	na	NYC	tubular	*CNGB1*	Comp Het	ar	
IRD068	RP		no	European	F	53	no	10/10	10/10	BS (BE)	extrafoveal AP (BE)	hypovolted (BE)	na	na	NYC,VI	peripheral deficit	*CNGB1*	Comp Het	ar	
IRD085	RP		no	European	F	57	no	5/10	1/10	BS,RA (BE)	AP,AR (BE)	extinct (BE)	na	na	NYC,VI	tubular	*CNGB1*	Hom	ar	
IRD032	nd IRD		no	European	M	2	no	na	NA	na	na	reduced amplitude C (BE)	na	na	NYS	na	*CNGB3*	Comp Het	ar	ACHM
IRD029	RP	familiar case	yes	Middle Asian	M	18	no	1/10	1/10	MA,RA,PIG (BE)	AP,AR,AM (BE)	extinct (BE)	abnormal (BE)	na	VI	central scotoma+peripheral deficit	*CRB1*	Hom	ar	
IRD030	RP		yes	Middle Asian	M	23	no	1/10	1/10	MA,RA,PIG (BE)	AP,AR,AM (BE)	extinct (BE)	abnormal (BE)	na	VI	central scotoma+peripheral deficit	*CRB1*	Hom	ar	
IRD031	RP		yes	Middle Asian	F	52	no	HM	HM	MA,RA,PIG (BE)	AP,AR,AM (BE)	extinct (BE)	abnormal (BE)	na	VI	central scotoma+peripheral deficit	*CRB1*	Hom	ar	
IRD035	LCA		no	European	F	24	no	HM	HM	salt and pepper, AV (BE)	AP (BE)	flat C (BE)	na	na	NYS	complete loss	*CRX*	Het	ad	
IRD008	RP		no	European	M	48	no	LP	1/10	RA (BE); MA,PIG (RE); BS (LE)	AP,AR,AM (BE)	extinct (BE)	na	na	NYC,VI	tubular	*PDE6B*	Hom	ar	
IRD013	RP		no	European	F	50	no	5/10	6/10	BS,RA (BE)	AP,AR (BE)	extinct (BE)	na	na	VI	central scotoma+peripheral deficit	*PDE6B*	Comp Het	ar	
IRD026	RP		yes	European	M	52	no	3/10	5/10	BS (BE)	extrafoveal AP (BE)	hypovolted (BE)	na	na	NYC,VI	tubular	*RHO*	Het	ad	
IRD016	RP		no	European	M	51	no	7/10	1/10	BS (BE)	extrafoveal AP (BE)	hypovolted (BE)	na	na			*ROM1*	Comp Het	ar	
IRD033	RP		no	European	M	18	no	LP	LP	AR, PIG (osteoblatic-like deposits), choroideremia (BE)	na	not detectable	na	na	NYC	na	*RP2*	Hem	xl	
IRD076	RP		no	European	M	63	no	3/10	5/10	BS (BE)	extrafoveal AP (BE)	hypovolted (BE)	na	na	NYC,VI	tubular	*RPE65*	Hom	ar	
IRD001	RP		no	European	F	37	no	LP	LP	MA,RA,PIG (BE)	AP,AR,AM (BE)	extinct (BE)	abnormal (BE)	na	NYS,VI	complete loss	*RPE65*	Comp Het	ad	
IRD074	LCA		yes	Middle Asian	M	9	no	HM	HM	MA,RA,PIG (BE)	AP,AR,AM (BE)	extinct (BE)	abnormal (BE)	na	NYS,VI	na	*RPE65*	Hom	ar	
IRD002	LCA		no	European	F	5	no	1/100	1/100	normal (BE)	normal (BE)	extinct (BE)	abnormal (BE)	na	NYS,VI	na	*RPGRIP1*	Comp Het	ar	
IRD012	RP		no	European	M	60	no	HM	1/100	BS,RA (BE)	AP,AR (BE)	extinct (BE)	na	na	NYC,VI	tubular	*RPGR*	Hem	xl	
IRD067	RP		no	European	M	2	no	2/10	3/10	AV, BS, AM (BE)	AP (BE), ERM (RE)	na	na	na	NYC	tubular	*RPGR*	Hem	xl	
IRD075	RP		no	European	M	37	no	4/10	7/10	BS,RA (BE)	AP,AR (BE)	extinct (BE)	na	na	NYC,VI	tubular	*RPGR*	Hem	xl	
IRD017	RP		no	European	M	33	no	3/10	5/10	BS,RA (BE)	AP,AR (BE)	extinct (BE)	na	na	NYC,VI	tubular	*RPGR*	Hem	xl	
IRD059	RP	familiar case	no	European	F	5	no	20/400	20/200	AV, BS, MA (BE)	AP,AR (BE), ERM (LE)	flat (BE)	na	na	PVL	tubular+ central scotoma	*TULP1*	Comp Het	ar	
IRD060	RP		no	European	F	2	no	20/100	LP	AV, BS, MA (BE)	AP,AR (RE)	flat (BE)	na	na	PVL	tubular+ central scotoma	*TULP1*	Comp Het	ar	
IRD041	RP		no	European	M	9	no	10/10	10/10	normal (BE)	normal (BE)	hypovolted (BE)	normal (BE)	na	NYC	peripheral deficit	*TULP1*	Comp Het	ar	
IRD007	USH		no	European	M	51	yes	1/100	2/10	BS,MA,RA,PIG (BE)	AP,AR,AM (BE)	extinct (BE)	na	na	NYC,VI	tubular	*USH2A*	Comp Het	ar	
IRD009	USH		no	European	F	53	yes	HM	1/100	BS,MA,RA,PIG (BE)	AP,AR,AM (BE)	extinct (BE)	na	na	NYC,VI	complete loss	*USH2A*	Comp Het	ar	
IRD021	RP		no	South American	F	46	no	9/10	9/10	BS,RA (BE)	AP,AR (BE)	extinct (BE)	na	na	NYC,VI	tubular	*USH2A*	Comp Het	ar	
IRD023	RP	familiar case	yes	European	F	63	yes	LP	1/10	BS,MA,RA,PIG (BE)	AP,AR,AM (BE)	extinct (BE)	na	na	NYC,VI	complete loss	*USH2A*	Comp Het	ar	USH
IRD024	RP		yes	European	M	55	yes	4/10	5/10	BS,MA,RA,PIG (BE)	AP,AR,AM (BE)	extinct (BE)	na	na	NYC,VI	tubular	*USH2A*	Comp Het	ar	USH
IRD038	RP		no	European	F	51	no	10/10	10/10	BS (BE)	normal (BE)	hypovolted (BE)	normal (BE)		NYC	peripheral deficit	*USH2A*	Comp Het	ar	
IRD084	USH		no	European	M	33	yes	10/10	10/10	BS (BE)	normal (BE)	hypovolted (BE)	normal (BE)		NYC	peripheral deficit	*USH2A*	Hom	ar	
IRD034	RP		no	European	F	48	no	10/10	10/10	salt and pepper (BE)	AP extramacular (BE)	flat (C and S), reduced amplitude (P) (BE)	na	normal	NYC	tubular	*USH2A*	Hom	ar	

AP, atrophy of the photoreceptor cell layer; AR, atrophy of the retinal pigment epithelium; AV, attenuation of the retinal vessels; BE, both eyes; BMD, Best macular Dystrophy; BS, bone spicules; C, combined; CVS, central visual loss; EOG, Electrooculography; ERG, Electroretinography; ERM, epiretinalmembrane;
Familiar case: the patients were from the same family; FF, fundus flavimaculatus; HM, hand motion; LCA, Leber Congenital Amaurosis; LE, left eye; LP, light perception; MA, macular atrophy; na, not available; nd IRD not defined Inerithed Retinal Dystrophy; NYC, nyctalopia; NYS, nystagmus; OCT, Optical coherence tomography;
P, photopic; PIG, pigmentary deposition; PVL, peripheral visual field loss; RA, retinal atrophy; RE, right eye; RP, Retinitis Pigmentosa; S, scotopic; STGD, Stargardt Disease; USH, Usher Syndrome; VI, Visual impairment;

Supplemental Table 2

Gene symbol	Gene title	Retinal diseases*
ABCA4	ATP-binding cassette transporter - retinal	recessive Stargardt disease, juvenile and late onset; recessive MD; recessive RP; recessive fundus flavimaculatus; recessive CORD
AIPL1	arylhydrocarbon-interacting receptor protein-like 1	recessive LCA; dominant CORD
ARL2BP	ADP-ribosylation factor-like 2 binding protein	recessive RP
BEST1	bestrophin 1	dominant MD, Best type; dominant vitreoretinochoroidopathy; recessive bestrophinopathy; recessive RP; dominant RP
C2orf71	chromosome 2 open reading frame 71	recessive RP
C8orf37	chromosome 8 open reading frame 37	recessive CORD; recessive RP with early macular involvement
CA4	carbonic anhydrase IV	dominant RP
CABP4	calcium binding protein 4	recessive congenital stationary night blindness; recessive congenital cone-rod synaptic disease; recessive LCA
CEP290	centrosomal protein 290 kDa	recessive Senior-Loken syndrome; recessive Joubert syndrome; recessive LCA; recessive Meckel syndrome
CERKL	ceramide kinase-like protein	recessive RP; recessive CORD with inner retinopathy
CLRN1	clarin-1	recessive Usher syndrome, type 3; recessive RP
CNGA1	rod cGMP-gated channel alpha subunit	recessive RP
CNGB1	rod cGMP-gated channel beta subunit	recessive RP
CNGB3	cone cyclic nucleotide-gated cation channel beta 3 subunit	recessive achromatopsia Pingelapese; recessive, progressive COD
CRB1	crumbs homolog 1	recessive RP with para-arteriolar preservation of the RPE (PPRPE); recessive RP; recessive LCA; dominant pigmented paravenous chorioretinal atrophy
CRX	cone-rod otx-like photoreceptor homeobox transcription factor	dominant CORD; recessive, dominant and de novo LCA; dominant RP
DHDDS	dehydrodolichyl diphosphate synthetase	recessive RP
ELOVL4	elongation of very long fatty acids protein	dominant MD, Stargardt-like; recessive spinocerebellar ataxia; recessive ichthyosis, quadriplegia and retardation
EYS	eyes shut/spacemaker (Drosophila) homolog	recessive RP
FAM161A	family with sequence similarity 161 member A	recessive RP
FSCN2	retinal fascin homolog 2, actin bundling protein	dominant RP; dominant MD
GUCA1B	guanylate cyclase activating protein 1B	dominant RP; dominant MD
GUCY2D	retinal-specific guanylate cyclase	recessive LCA; dominant CORD
IDH3B	NAD(+)-specific isocitrate dehydrogenase 3 beta	recessive RP
IMPDH1	inosine monophosphate dehydrogenase 1	dominant RP; dominant LCA
IMPG2	interphotoreceptor matrix	recessive RP

	proteoglycan 2	
IQCB1	IQ motif containing B1 protein	recessive Senior-Loken syndrome; recessive LCA
KCNJ13	inwardly-rectifying potassium channel subfamily J member 13	dominant vitreoretinal degeneration, snowflake; recessive LCA
KLHL7	kelch-like 7 protein (Drosophila)	dominant RP
LCA5	lebercilin	recessive LCA
LRAT	lecithin retinol acyltransferase	recessive RP, severe early-onset; recessive LCA
MAK	male germ-cell associated kinase	recessive retinits pigmentosa
MERTK	c-mer protooncogene receptor tyrosine kinase	recessive RP; recessive rod-COD, early onset
NMNAT1	nicotinamide nucleotide adenylyltransferase 1	recessive LCA
NR2E3	nuclear receptor subfamily 2 group E3	recessive enhanced S-cone syndrome (ESC); recessive RP in Portuguese Crypto Jews; recessive Goldmann-Favre syndrome; dominant RP; combined dominant and recessive retinopathy
NRL	neural retina lucine zipper	dominant RP; recessive RP
OFD1	oral-facial-digital syndrome 1 protein	Jobert syndrome; orofaciodigital syndrome 1, Simpson-Golabi-Behmel syndrome 2; X-linked RP, severe
ORP1	RP1 protein	dominant RP; recessive RP
OTX2	orthodenticle homeobox 2 protein	dominant LCA and pituitary dysfunction; recessive microphthalmia
PDE6A	cGMP phosphodiesterase alpha subunit	recessive RP
PDE6B	rod cGMP phosphodiesterase beta subunit	recessive RP; dominant congenital stationary night blindness
PDE6G	phosphodiesterase 6G cGMP-specific rod gamma	recessive RP
PRCD	progressive rod-cone degeneration protein	recessive RP
PROM1	prominin 1	recessive RP with macular degeneration; dominant Stargardt-like MD; dominant MD, bull's-eye; dominant CORD
PRPF3	pre-mRNA processing factor 3	dominant RP
PRPF31	pre-mRNA processing factor 31	dominant RP
PRPF6	pre-mRNA processing factor 6	dominant RP
PRPF8	pre-mRNA processing factor 8	dominant RP
PRPH2	peripherin 2	dominant RP; dominant MD; digenic RP with ROM1; dominant adult vitelliform MD; dominant CORD; dominant central areolar choroidal dystrophy; recessive LCA
RBP3	retinol binding protein 3, interstitial	recessive RP
RD3	RD3 protein	recessive LCA
RDH12	retinol dehydrogenase 12	recessive LCA with severe childhood retinal dystrophy; dominant RP
RGR	RPE-retinal G protein-coupled receptor	recessive RP; dominant choroidal sclerosis
RHO	rhodopsin	dominant RP; dominant congenital stationary night

		blindness; recessive RP
RLBP1	retinaldehyde-binding protein 1	recessive RP; recessive Bothnia dystrophy; recessive retinitis punctata albescens; recessive Newfoundland rod-COD
ROM1	retinal outer segment membrane protein 1	dominant RP; digenic RP with PRPH2
RP1	RP1 protein	dominant RP; recessive RP
RP1L1	retinitis pigmentosa 1-like protein 1	dominant occult MD; recessive RP
RP2	retinitis pigmentosa 2 (X-linked)	X-linked RP; X-linked RP, dominant
RP9	RP9 protein or PIM1-kinase associated protein 1	dominant RP
RPE65	retinal pigment epithelium-specific 65 kD protein	recessive LCA; recessive RP; dominant RP with choroidal involvement
RPGR	retinitis pigmentosa GTPase regulator	X-linked RP, recessive; X-linked RP, dominant; X-linked COD 1; X-linked atrophic MD, recessive
RPGRIP1	RP GTPase regulator-interacting protein 1	recessive LCA; recessive CORD
SAG	arrestin (s-antigen)	recessive Oguchi disease; recessive RP
SEMA4A	semaphorin 4A	dominant RP; dominant CORD
SNRNP200	small nuclear ribonucleoprotein 200kDa (U5)	dominant RP
SPATA7	spermatogenesis associated protein 7	recessive LCA; recessive RP, juvenile
TOPORS	topoisomerase I binding arginine/serine rich protein	dominant RP
TTC8	tetratricopeptide repeat domain 8	recessive Bardet-Biedl syndrome; recessive RP
TULP1	tubby-like protein 1	recessive RP; recessive LCA
USH2A	usherin	recessive Usher syndrome, type 2a; recessive RP
ZNF513	zinc finger protein 513	recessive RP

COD: cone dystrophy; CORD: cone-rod dystrophy; LCA: Leber congenital amaurosis;
MD: macular dystrophy; RP: retinitis pigmentosa
* as listed in Retnet, the Retinal Information Network (https://sph.uth.edu/retnet/home.htm)

Supplemental Table 3

Supplementary Table 3. Primers used for PCR and Sanger sequencing

Primers	Sequence	PCR product
CNGB3_13F	5'-ACAGGCTTCAACCTATCACCGGAA-3'	548bp
CNGB3_13R	5'-ACCCAGACTGTCTCTGGAGTGGCA-3'	
RPGR_F1	5'-TTCCCATTTCCCTGTGTGTTAGT-3'	1363bp
RPGR_R1	5'-GTAGTTCAGGAGCAGAACACTGG-3'	
RPGR_F2	5'-CTGTCTCCTGATACTTCCCCTCT-3'	1339bp
RPGR_R2	5'-TCAGATGACCTTACAGACAAAGCA-3'	
Additional primers for sequencing		
RPGR_F3	5'-CTCCTTCCTCCCCTTCCACCTCCCCTTCCA-3'	
RPGR_R3	5'-AGTGGAGGGAGAACGTGAAA-3'	
RPGR_R4	5'-GGAGGGAGGGGAAGTAGAGGGAGGGGAAGT-3'	
